# Effect of Electrical Stimulation on PC12 Cells Cultured in Different Hydrogels: Basis for the Development of Biomaterials in Peripheral Nerve Tissue Engineering

**DOI:** 10.3390/pharmaceutics15122760

**Published:** 2023-12-12

**Authors:** Yusser Olguín, Mónica Selva, Diego Benavente, Nicole Orellana, Ivan Montenegro, Alejandro Madrid, Diego Jaramillo-Pinto, María Carolina Otero, Tomas P. Corrales, Cristian A. Acevedo

**Affiliations:** 1 Departamento de Química y Medio Ambiente, Universidad Técnica Federico Santa María, Avenida España 1680, Valparaíso 2390123, Chile; 2 Centro Científico y Tecnológico de Valparaíso (CCTVal), Universidad Técnica Federico Santa María, Avenida España 1680, Valparaíso 2390123, Chile; diego.benavente@usm.cl (D.B.); cristian.acevedo@usm.cl (C.A.A.); 3 Centro de Biotecnología, Universidad Técnica Federico Santa María, Avenida España 1680, Valparaíso 2390123, Chile; movaseza@gmail.com (M.S.); nicole.orellana@usm.cl (N.O.); tomas.corrales@usm.cl (T.P.C.); 4 Centro de Investigaciones Biomédicas, Escuela de Obstetricia, Facultad de Medicina, Universidad de Valparaíso, Angamos 655, Reñaca, Viña del Mar 2520000, Chile; ivan.montenegro@uv.cl; 5 Laboratorio de Productos Naturales y Síntesis Orgánica (LPNSO), Departamento de Ciencias y Geografía, Facultad de Ciencias Naturales y Exactas, Universidad de Playa Ancha, Avda. Leopoldo Carvallo 270, Playa Ancha, Valparaíso 2390123, Chile; alejandro.madrid@upla.cl; 6 Departamento de Física, Universidad Técnica Federico Santa María, Avenida España 1680, Valparaíso 2390123, Chile; diego.jaramillop@usm.cl; 7 Millenium Nucleus in NanoBioPhysics (NNBP), Universidad Técnica Federico Santa María, Valparaíso 2390123, Chile; 8 Escuela de Química y Farmacia, Facultad de Medicina, Universidad Andres Bello, Republica 252, Santiago 8370071, Chile; maria.otero@unab.cl

**Keywords:** peripheral nerve tissue engineering, electrical stimulation, hydrogels, PC12 cells

## Abstract

Extensive damage to peripheral nerves is a health problem with few therapeutic alternatives. In this context, the development of tissue engineering seeks to obtain materials that can help recreate environments conducive to cellular development and functional repair of peripheral nerves. Different hydrogels have been studied and presented as alternatives for future treatments to emulate the morphological characteristics of nerves. Along with this, other research proposes the need to incorporate electrical stimuli into treatments as agents that promote cell growth and differentiation; however, no precedent correlates the simultaneous effects of the types of hydrogel and electrical stimuli. This research evaluates the neural differentiation of PC12 cells, relating the effect of collagen, alginate, GelMA, and PEGDA hydrogels with electrical stimulation modulated in four different ways. Our results show significant correlations for different cultivation conditions. Electrical stimuli significantly increase neural differentiation for specific experimental conditions dependent on electrical frequency, not voltage. These backgrounds allow new material treatment schemes to be formulated through electrical stimulation in peripheral nerve tissue engineering.

## 1. Introduction

Tissue engineering has emerged as a promising field in regenerative medicine, especially in restoring and replacing damaged or degenerated tissues [1]. Among body tissues, peripheral nerve tissue (PNT) presents a unique set of challenges and opportunities. Unlike many other tissues, the PNT can inherently regenerate; however, in extensive injuries, this regeneration is often insufficient, resulting in persistent loss of tissue function [2]. In this context, the development of biomaterials plays a fundamental role by establishing themselves as synthetic extracellular matrices that provide physical support and biochemical signals sufficient for cell growth and differentiation [3,4].

Within this realm, hydrogels are beautiful materials in peripheral nerve tissue engineering because, being highly hydrated and permeable, they closely mimic the viscoelastic nature of native tissues as the nerves [5]; in addition, such characteristics make them particularly apt for cell encapsulation and controlled delivery of bioactive factors, thus facilitating nerve tissue regeneration [6,7]. Specifically, hydrogels such as gelatin methacrylate (GelMA), polyethylene glycol diacrylate (PEGDA), collagen, and alginate have shown potential in nerve tissue engineering applications due to their biocompatibility and tunable mechanical properties [7,8,9,10,11].

However, the nerve cellular environment is not solely dictated by biochemical cues; electrical signals play a foundational role, where the physiological importance of two types of electrical signals is recognized: the low resistance intracellular signals propagated between two neurons through gap junctions and the electrical signals between neurons without contact between them derived from extracellular electric fields [12]. Numerous studies have demonstrated that electrical stimulation can promote cell proliferation, neurite elongation, and axonal growth direction [13,14,15]. Thus, combining biomaterials, such as the hydrogels above, with electrical stimuli might offer a synergistic strategy, enhancing PNT regeneration effectively.

The present investigation evaluates the interaction between electrical stimulation and neural differentiation of PC12 cells cultured on different hydrogels. This approach aims to relate the effects of hydrogel properties and electrical stimulation as relevant parameters for the generation of future therapeutic alternatives in peripheral nerve tissue engineering.

## 2. Materials and Methods

### 2.1. Reagents

The reagents used in this study were: penicillin–streptomycin (AB), horse serum (HS), fetal bovine serum (FBS), collagen I, rat tail, phosphate buffer saline (PBS), and modified RPMI-1640 ATCC (Gibco TM); porcine skin gelatin, methacrylic anhydride, 2-hydroxy-4′-(2-hydroxyethoxy)-2-methylpropiophenone (Irgacure), 2-hydroxy-2-methylpropiophenone (Darocur 1173), alginate, Poly(ethylene glycol) diacrylate (PEGDA) and nerve growth factor (NGF) and WST-1 assay kit (Sigma-Aldrich; Merck KGaa, Germany). Other reagents were purchased for these experiments with sufficient quality.

### 2.2. PC12 Cell Culture, Differentiation and Viability

PC12 cells were cultured in RPMI-1640 ATCC modified medium supplemented with 10% HS, 5% FBS, 100 u/mL penicillin, and 100 mg/L streptomycin. PC12 cells were maintained at 37 °C in a humidified incubator with 5% CO_2_. The differentiation medium consisted of RPMI-1640 ATCC-modified medium supplemented with 2% HS, penicillin–streptomycin 100 ug/mL, and 50 ng/mL NGF. Cell viability and proliferation were assessed with the WST1 assay in 96-well plate format following standard protocol [16]. PC12 cells (2 × 10^5^ cells/mL) were seeded on the different hydrogels for cell assays (this amount was adjusted due to differences in cell adhesion). Neural differentiation was determined by microphotographs measuring neurite extension and the percentage of neurites per cell using the ImageJ single neurite tracer system^®^. To measure the extent of the neurites, manually set the origin and end point of each neurite for the software to estimate the measurement. On the other hand, to determine the percentage of cells expressing neurites, the ratio between the total number of cells present in microphotography and the number of cells showing at least one neurite is calculated. For each measurement, four microphotographs of each culture were used in triplicate.

### 2.3. Preparation of Hydrogels and Surface Modification of Culture Systems

Collagen: For the modified protocol, the collagen solution was prepared by dissolving 1.5 mL of collagen (Collagen I) in 30 mL of 20 mM acetic acid. A total of 2 mL of the solution was deposited in each well of the six-well plate, left to incubate for two hours, and then washed three times with PBS pH 7.4 [17].

PEGDA: For the modified protocol, the hydrogel was prepared by mixing 1 g of PEGDA and 31 mg of Darocur 1173 (photoinitiator), dissolved in 10 mL of HEPES. A total of 1.5 mL of solution was deposited in each well of a six-well culture plate and exposed to UV radiation (0.4 joules), the wells were washed three times with PBS pH 7.4 [18].

Alginate: For the modified protocol, the hydrogels were prepared by ionic polymerization by mixing 800 μL of an alginate solution and 1 mL of 100 μM CaCl_2_. A total of 2 mL of the mixture is placed in each well of the six-well culture dish, allowed to rest for 30 min, and washed 3 times with PBS pH 7.4. The alginate solution is prepared by dissolving 0.5 g of alginate in 50 mL of culture medium (RPMI 10%, HS 5% FBS, 1% AB) for a final concentration of 0.01% *w*/*v* [19].

GelMA: The synthesis of methacrylated gelatin (GelMA) was prepared from a modified protocol by reaction of gelatin with methacrylic anhydride. From a 10% (*w*/*v*) gelatin solution in PBS (pH 7.5) at 50° C, methacrylic anhydride was added slowly (0.2 mL/min) under vigorous stirring until a 20% *v*/*v* gelatin solution was formed. The resulting solution was diluted 1:2 in PBS and dialyzed for 3 days in Milli-Q water at 40° C. The product was freeze-dried to obtain a powder in varying amounts [20]. Treatment of the culture surfaces was performed by dissolving 0.1 g of GelMA in 2 mL of PBS, plus 500 μL of culture medium and 100 μL of Irgacure (photoinitiator) 4:5:1, 1.5 mL of the mixture was deposited per well in six-well plates and irradiated with UV 0.2 joule.

### 2.4. Electrical Stimulation

**Figure 1:**
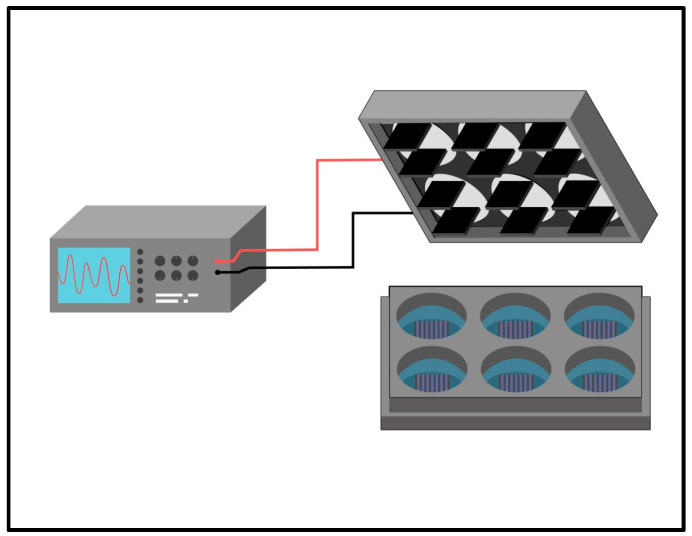
Graphical representation of culture system and electrical stimulation in six-well plate format.

As shown in Figure 1, the electrical stimulation system consists of an arbitrary wave generator connected to a stimulation module composed of parallel-positioned carbon electrodes adapted for six-well plates. The cells are cultured in a six-well plate, and for the moment of electrical stimulation, the plastic lid is removed, and the described artifact is incorporated. The carbon electrodes touch the culture medium, and the stimulation begins. Plates previously treated with hydrogels were subjected to electrical stimulation by four types of stimuli, ES1 (200 mV 50 Hz), ES2 (400 mV 50 Hz), ES3 (200 mV 100 Hz), and ES4 (400 mV 100 Hz), for two hours per day of experimentation [21,22].

### 2.5. AFM Measurements

Two different AFMs were used to study the roughness and mechanical properties of four hydrogel solutions: collagen, alginate, PEGDA, and GELMA. The collagen sample was measured using a Flex AFM (Nanosurf Inc., Basel, Switzerland), while alginate, PEGDA, and GelMA were measured with a NanoWizard3 (JPK, Berlin, Germany). The AFM chips used for this research were qp-BioAC by NanoSensors (Watsonville, CA, USA). These AFM chips have three different cantilevers with different elastic constants and dimensions. Each cantilever has a tip radius of around 10 nm and a Uniqprobe coating that consists of a reflective gold layer. This coating is applied to the backside of the cantilever, which faces the detector. This coating helps reduce the bending of the AFM cantilever and its chemical stability when measuring in liquid environments. In particular, the AFM measurements were performed in a PBS solution. 

For the four samples, we use the same measurement protocol, which is described as follows. First, we use AFM tapping mode to make topographical images of the hydrogel surface [23]. Then, we perform force–volume spectroscopy to obtain the nanomechanical properties over the previously imaged region [24]. Calibration of the tip’s spring constants is made using the thermal noise method [25]. Force–indentation curves generated by this method are then fitted using the Hertz, model obtaining the surface’s Young’s modulus as a fitting parameter [26].

### 2.6. Statistical Analysis

The experimental design included 9 independent determinations per electrical stimulus condition and hydrogel type. The plotted results are represented with the mean and standard deviation (SD). ANOVA performed statistical tests plotted as (* *p* < 0.05; ** *p* < 0.01 and *** *p* < 0.001). Post hoc Tukey multiple comparisons tests were performed to determine interactions between factors affecting the dependent variable [27,28]. Analysis of variances and their ratios were calculated by Tukey post hoc tests using OriginLab^®^ software (OriginPro 2021).

## 3. Results and Discussion

### 3.1. PC12 Cells Proliferation and Viability on Hydrogels

**Figure 2:**
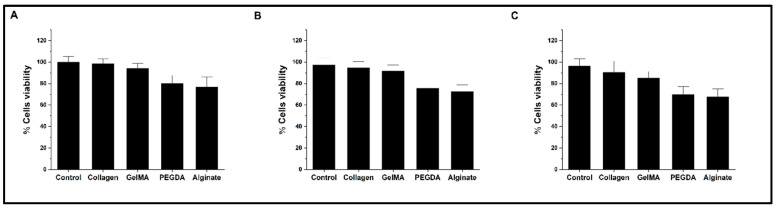
Bar graph of cell viability levels by WST1 assay. (**A**–**C**) represents the results of measurements at 24, 48, and 72 h, respectively.

The proliferation and viability of PC12 cells, measured by WST1 assay, show the effect of the surfaces coated with the different hydrogels in independent experiments: 24 h, 48 h, and 72 h, shown in Figure 2A–C, respectively. In the assay, the number of cells was adjusted due to differences in cell adhesion, which is significantly lower with alginate and PEGDA. The cell viability results show no significant differences for plates coated with collagen and GelMA compared to the control plate without hydrogel. In contrast, experiments with plates coated with PEGDA and alginate show a progressive reduction of viability (75.5 ± 3.8% and 72.7 ± 3.6% at 48 h and 69.8 ± 4.5% and 67.5 ± 4.7% at 72 h, respectively). For these assays, the measurement after 4 h of incubation of the WST1-treated cultures was used as time zero, which allows the establishment of an inference base that better fits the experimental conditions.

Hydrogels are often used in cell cultures due to their ability to mimic elements of native extracellular matrices, providing mechanical similarity, resembling different soft tissues, and providing a chemical configuration that favors cell adhesion and protein binding [29]. Notably, the PC12 cell line to differentiate and express 2D neurites must be cultured on collagen hydrogels, which provide favorable physical characteristics and a surface configuration that increases cell adhesion [30]. Our results show that for PC12 cell differentiation, the characteristics of GelMA, like collagen, are favorable. GelMA is recognized as a hydrogel with good surface properties that favor cell adhesion [31]. However, with alginate and PEGDA, these properties should be optimized by modifying their concentrations or by incorporating molecules that increase the adhesion of proteins that are part of the incorporation of cells on the surfaces [32,33]; this could explain the results obtained.

### 3.2. Differentiation of PC12 Cells in Different Hydrogels

**Figure 3:**
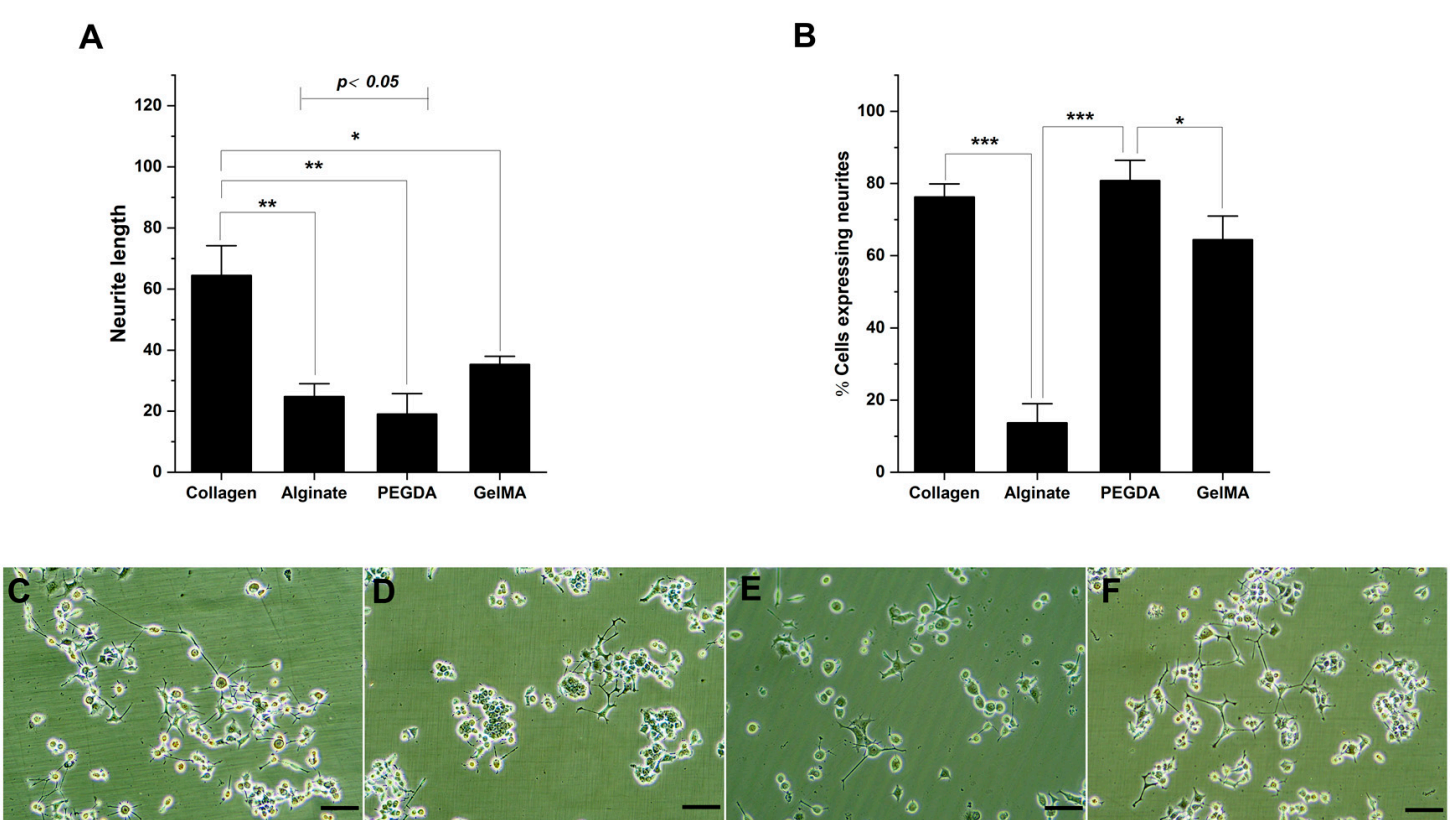
Neural differentiation of PC12 cells according to topography. (**A**). Bar graph of neurite length measurements. (**B**). Bar graph of the percentage of cells expressing neurites. (**C**–**F**). Representative microphotographs of PC12 cells in collagen, alginate, PEGDA, and GelMA, respectively. (* *p* < 0.05; ** *p* < 0.01 and *** *p* < 0.001).

The process of neural differentiation of PC12 cells depends on several factors, including the surface characteristics of the substrates where they are cultured [34]. In this sense, neural differentiation was evaluated in the different hydrogels. For this assay, the methodology commonly used for PC12 cell differentiation was homologized, which includes modification of the serum composition in the culture medium, addition of NGF, and treatment of the culture surfaces using collagen; for our assays, the latter was exchanged for the hydrogels studied. Morphological changes in neural differentiation of PC12 cells are related to neurite outgrowth. The magnitude of differentiation in an analysis of the cultured cell population is evidenced by the percentage of cells expressing neurites and the average neurite lengths. In these assays, due to the lower cell adhesion in alginate and PEGDA, the number of seeded cells was increased to achieve homogeneous cultures of adherent cells. The results show a significant effect of the hydrogels used on neural differentiation. Specifically, regarding neurite length, in cell cultures where collagen was used, the lengths were more significant on average, 64.5 ± 7.1 mm, followed by GelMA 35.3 ± 5.7 mm, alginate 24.8 ± 3.4 mm and PEGDA 19.1 ± 4.5 mm (Figure 3A). This behavior was not correlatable with the measurement of the percentage of cells expressing neurite; these results show that for cultures with PEGDA, the percentage was 80.8 ± 3.8%, for collagen 76.3 ± 4.7%, for GelMA 64.5 ± 5.1%, and alginate 13.7 ± 2.8% (Figure 3B). Among the cultures analyzed, the significant differences are expressed in asterisks in the bar graphs described in materials and methods. The microphotographs in Figure 3C–F show typical behaviors for the different cultures analyzed in this study.

**Figure 4:**
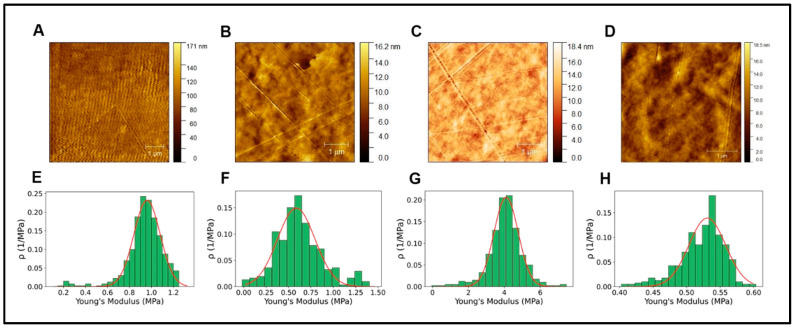
(**A**–**D**). AFM topographical image alginate, collagen, GelMA, and PEGDA, respectively. (**E**–**H**). Young’s modulus histograms of alginate, collagen, GelMA, and PEGDA, respectively.

Cell behavior depends on the substrate surfaces’ physical, chemical, and topographical characteristics, creating a favorable interaction dependent on the prior binding of specific serum proteins to the surfaces [35,36]. In the differentiation of PC12 cells, as in other similar cell types, the extension of neurites is closely related to the mechanical stress and stiffness of the substrates where they proliferate and differentiate, affecting axonal strain [37,38]. However, these conditions show complex correlations where several factors influence the behavior of neural development in an indeterminate and difficult-to-predict way [39,40,41]. To determine the existence of a statistical correlation between the topographical characteristics of the hydrogels and the neural differentiation of PC12 cells, surface characterization was carried out using AFM; precisely, Young’s modulus and roughness were measured (Table 1 and Figure 4). Considering the results obtained from the young module, these are similar to those reported [42,43], but it is not possible to infer that the differences in the neural differentiation behavior shown in Figure 2 are significantly due to the stiffness of the different hydrogels, whose values are not correlatable with cells behavior.

Roughness is another crucial parameter in defining the topographical characteristics of the surfaces used for cell growth [44]. Roughness affects the adsorption of surface-binding proteins, which play an elemental role in cell adhesion [45]; however, the significant magnitude of the effects of changes in roughness is often evaluated in a particular way, mainly because changes in cell behavior respond to multiple variables [46]. There could be a threshold or limit of roughness dimensions that affect the magnitude of cellular responses; this limit would depend on cell types and culture conditions [47].

Roughness is usually measured to determine two highly relevant parameters, the average surface roughness (Ra) and mean square roughness (RMS). Ra represents the arithmetic mean of the absolute values of the profile height deviations from the mean line recorded within the evaluation length. In contrast, RMS represents the mean square mean of the profile height deviations from the mean line recorded within the evaluation length [48]. 

The results expressed in Table 1 and Figure 4 show characteristic roughness lengths for these hydrogels used in the present investigation, particularly for collagen [49], GelMa [50] and alginate [51]; however, for hydrogels formed by PEGDA, the roughness results were significantly lower than those previously reported, as a unique contributor of the hydrogel [52], as well as when PEGDA was crosslinked with other polymers [53,54]. These roughness variations are related to the level of crosslinking and, with it, the magnitude of ultraviolet radiation in the hydrogel-forming process, which was higher in the preparations of the present investigation. In general, the roughness levels correlate negatively with the level of crosslinking of hydrogels [55], where increasing the level of crosslinking increases the surface stiffness and reduces the level of roughness [56]. PC12 cells demonstrate positive adhesion-related behavior when surfaces exhibit small roughness ranges or even at the nanometer level. Surfaces with minor roughness provide focal adhesion points for PC12 cells to bind, providing directional cues for growth and differentiation [57,58]. Our results fail to correlate effectively in all experimental conditions; a roughness and stiffness modulation test could provide sufficient background to determine with certainty the significance of the topographical characteristics in cellular behavior, which could be compared with the chemical characterization of the surfaces, which is outside the scope of the present study.

### 3.3. Differentiation of PC12 Cells: Effect of Electrical Stimulation

Electrical stimulation is considered a central element in developing biomaterials due to the different precedents demonstrating the relationship between the activation of different intracellular signaling pathways and the positive effect on cell behavior [59]. In general, hydrogels tend to have poor electrical conduction compared to culture media; due to this, several alternatives have been evaluated to improve these properties through different strategies, especially in 3D cultivation, such as chemical modification and doping of polymers, the addition of metallic nanoparticles or the addition of carbon nanostructures, however, these alternatives modify the biological and toxicological properties, which could limit the use of these hydrogels [60,61,62,63,64].

In the present research for the different culture systems with GelMA, collagen, alginate, and PEGDA hydrogels, the effect of electrical stimulation was evaluated in independent experiments based on described methods. The stimulation conditions correspond to: ES1 (200 mV 50 Hz); ES2 (400 mV 50 Hz); ES3 (200 mV 100 Hz) and ES4 (400 mV 100 Hz). The results are shown separately for each hydrogel.

**Figure 5:**
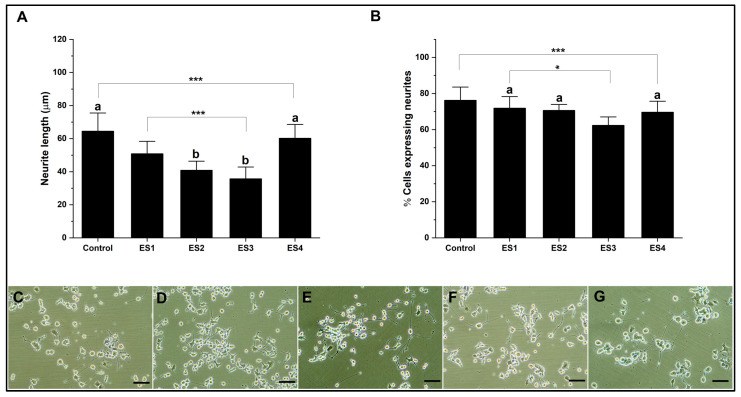
Neural differentiation in collagen. (**A**). Bar graph of neurite length. (**B**). Bar graph of the percentage of cells expressing neurites. (**C**–**G**). Representative micrographs of control, ES1, ES2, ES3, and ES4, respectively. (Reference bar 50 μm). (Lowercase letters above the error bars indicate statistical similarity between groups). (* *p* < 0.05 and *** *p* < 0.001).

Figure 5 shows the effect of electrical stimulation on neural differentiation of PC12 cells cultured in collagen. The results show that the length of neurites concerning the control is significantly lower for conditions ES1, ES2, and ES3, while for ES4, the levels are comparable. Regarding the percentage of cells expressing neurites, electrical stimulation does not show significant differences in the ES1 condition. At the same time, it is significantly lower for the ES2, ES3, and ES4 conditions concerning the control. The effects on neural differentiation in PC12 cells related to electrical stimulation are insignificant or appear negative when cultures are grown on collagen. This situation is related to the low electrical conductivity of collagen, so several reports indicate the need to modify the hydrogel to improve this property [65,66].

**Figure 6:**
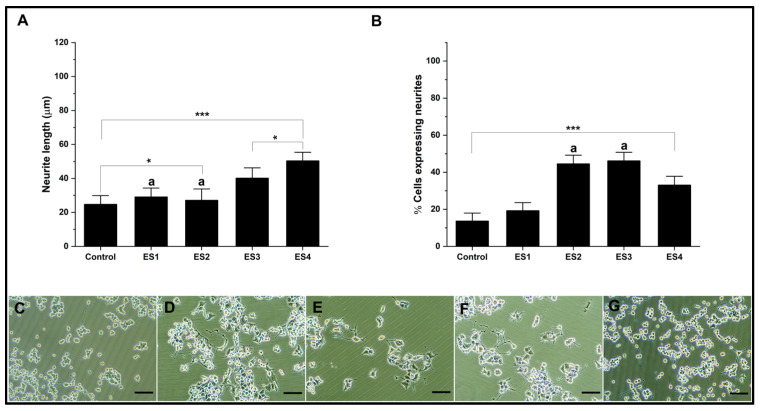
Neural differentiation in alginate. (**A**). Bar graph of neurite length. (**B**). Bar graph of percentage of cells expressing neurites. (**C**–**G**). Representative micrographs of control, ES1, ES2, ES3, and ES4, respectively. (Reference bar 50 μm). (Lowercase letters above the error bars indicate statistical similarity between groups). (* *p* < 0.05 and *** *p* < 0.001).

On the other hand, the electrical stimulation results on cultures in alginate hydrogels show a significant increase in the length of neurites concerning the control, mainly associated with conditions ES3 and ES4 (Figure 6A). Regarding the percentage of cells expressing neurites, the results show that electrical stimulation conditions significantly favor neural differentiation, mainly associated with ES2 and ES3 conditions (Figure 6B). Representative images can be seen in Figure 6C–G.

The results show a significant increase in neural differentiation in alginate hydrogels without the need to modify the conductive properties of the hydrogel. Other investigations show results under other stimulation conditions but with the need to modify the electrical conduction characteristics mainly in 3D cultures, for example, by generating hybrids with polypyrrole or carbon nanotubes, which ostensibly improves the capabilities of hydrogels to promote neural differentiation using electrical stimulation [67,68]. 

**Figure 7:**
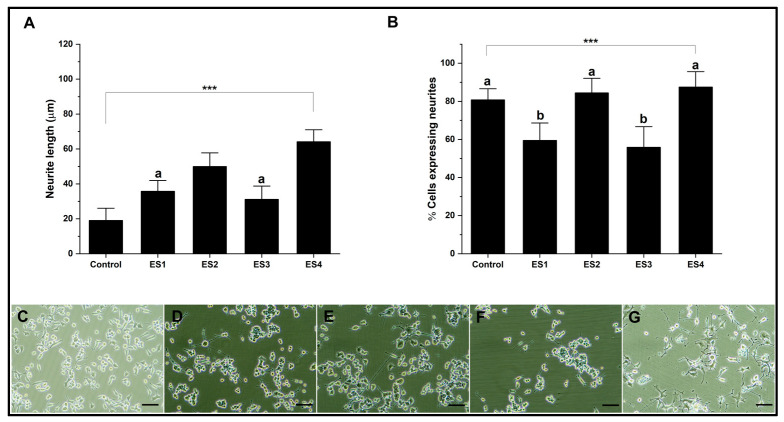
Neural differentiation in PEGDA. (**A**). Bar graph of neurite length. (**B**). Bar graph of percentage of cells expressing neurites. (**C**–**G**). Representative micrographs of control, ES1, ES2, ES3, and ES4, respectively. (Reference bar 50 μm). (Lowercase letters above the error bars indicate statistical similarity between groups). (*** *p* < 0.001).

The results of experiments performed on PEGDA show a significant effect of electrostimulation on neural differentiation (Figure 7). Graph A indicates that electrical stimulation, especially under the ES4 condition, increases neurite length compared to the control. On the other hand, graph B provides insight into the proportion of cells that differentiate and express neurites. The ES2 and ES4 stimulation conditions show a high level of neurite expression, similar to the control. Under these conditions, neural differentiation is subject to a specific frequency independent of voltage. Several precedents demonstrate the dependence of electrical frequency on cellular activity, which promotes proliferation and differentiation. These investigations suggest establishing a delicate balance between electrical conditions to promote differentiation, which depends on several factors in the culture, such as cell type [69,70,71].

**Figure 8:**
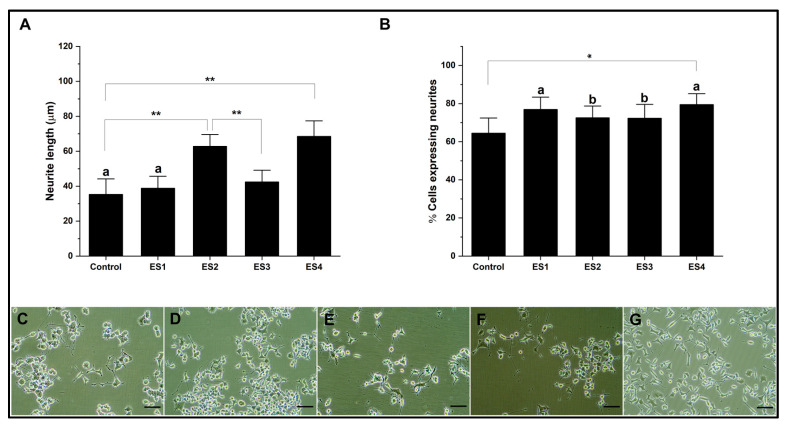
Neural differentiation in GelMA. (**A**). Bar graph of neurite length. (**B**). Bar graph of the percentage of cells expressing neurites. (**C**–**G**). Representative micrographs of control, ES1, ES2, ES3, and ES4, respectively. (Reference bar 50 μm). (Lowercase letters above the error bars indicate statistical similarity between groups). (* *p* < 0.05 and ** *p* < 0.01).

Finally, the electrical stimulation results on GelMa cultures show increased neural differentiation (Figure 8). Regarding neurite length, for ES2 and especially ES4 conditions, higher levels of neurite length are shown, a trend similar to that observed with PEGDA and electrical frequency dependence. Regarding the percentage of cells expressing neurites, all electrical stimulation conditions significantly increase concerning the control. Our results show the capacity of GelMa as a hydrogel capable of promoting neural differentiation efficiently in conjunction with electrical stimulation without modifying the polymer, which is fundamental and necessary, especially in three-dimensional cultures [72,73].

### 3.4. Statistical Analysis

**Figure 9:**
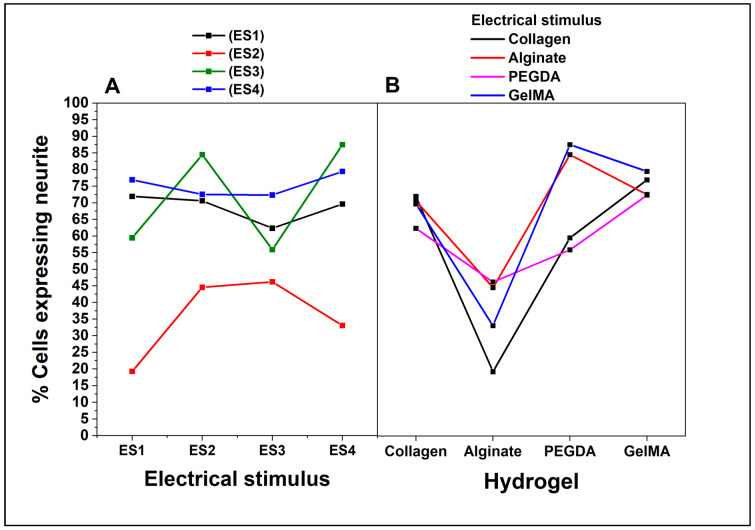
ANOVA Tukey interaction plot of percentage of cells expressing neurites. Effect of factors on the percentage of cells expressing neurites. (**A**). Type of electrical stimulation. (**B**). Type of hydrogel.

The results were analyzed using a multiple pairwise comparisons ANOVA test, considering two factors: type of hydrogel and type of electrical stimulus, each with four different levels. Figure 9 shows the ANOVA interaction plot using the percentage of cells expressing neurites as a dependent variable. Regarding the interaction between the hydrogel and electrical stimulus, Figure 9A, the cells in collagen show a relatively stable percentage of expression throughout the four electrical stimuli, approximately 80%. The cells have a markedly lower response to alginate than the other hydrogels. Although there is a slight increase in ES3, the percentage of expression is still considerably lower, below 40%. Meanwhile, for PEGDA and GelMA, similar response patterns are shown in these two hydrogels across electrical stimuli, although GelMA generally has a slightly higher percentage of neurite expression. Notably, both hydrogels peak at ES3, indicating that this specific stimulus potentiates neurite expression in cells cultured on these hydrogels.

The results of the interaction between the hydrogel and the dependent variable (Figure 9B) show how the dependent variable, the percentage of cells expressing neurites, varies as a function of the hydrogel. In this regard, collagen has the highest percentage, followed closely by GelMA, while alginate has the lowest percentage, consistent with its low response in the electrical stimulus plot. Finally, PEGDA shows an intermediate response. The overall interaction interpretation shows that the response of PC12 cells in terms of neurite expression depends not only on the type of hydrogel but also on the type of electrical stimulus to which they are subjected. While collagen appears to be the most conducive hydrogel for neurite expression under all conditions, GelMA and PEGDA show a particularly favorable response to the ES3 electrical stimulus. In contrast, alginate consistently shows a low ability to promote neurite expression, regardless of the electrical stimulus.

**Figure 10:**
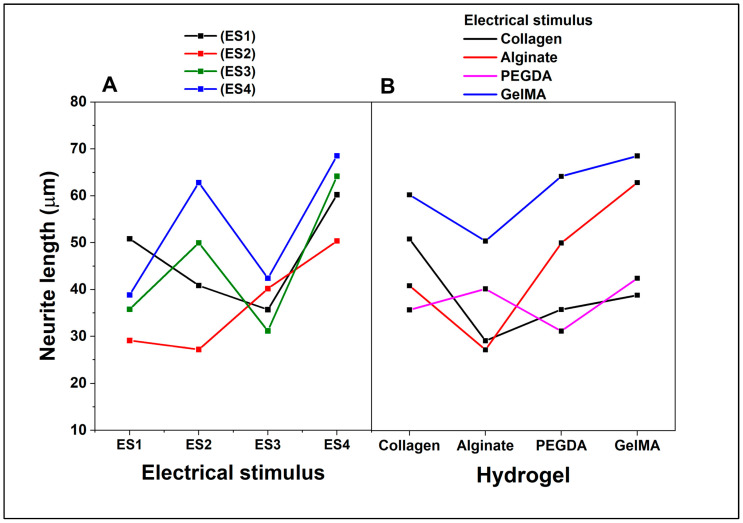
ANOVA Tukey interaction plot of length of neurites. Effect of factors on the percentage of cells expressing neurites. (**A**). Type of electrical stimulation. (**B**). Type of hydrogel.

On the other hand, ANOVA interaction analysis considering neurite length as a dependent variable (Figure 10) shows that in the interaction between the hydrogel and electrical stimulus when cells are cultured on collagen, the neurite length fluctuates along the electrical stimuli, with a peak in ES3 and a valley in ES4. In alginate, the cells show a consistently low neurite length, regardless of electrical stimulus, and remain below the other hydrogels. For the experiments with PEGDA, a noticeable peak is shown at ES2 and a valley at ES3, while GelMA neurite length increases with ES2 and ES3, the latter being the highest peak among all hydrogels at ES3.

When analyzing the interaction between the hydrogel and the dependent variable (Figure 10B), it is depicted how neurite length varies directly as a function of hydrogel without a specific electrical stimulus. Collagen and PEGDA have similar lengths, while alginate has the shortest length and GelMA has the longest. As an overall analysis, the length of neurites expressed by PC12 cells is influenced by the type of hydrogel to which they are cultured and the type of electrical stimulus to which they are subjected. GelMA is particularly conducive to promoting increased neurite length, especially under ES3 electrical stimulus. Alginate consistently shows a low ability to promote neurite extension under all electrical stimulus conditions. Cells in collagen and PEGDA show mixed responses depending on the electrical stimulus, but generally, they tend to have longer neurite lengths than alginate but shorter than GelMA.

## 4. Conclusions

The expression of neurites in PC12 cells is influenced by both the type of hydrogel and the type of electrical stimulus. Collagen consistently appears to be the most conducive hydrogel for promoting neurite expression across all conditions, with a stable high percentage of expression. GelMA and PEGDA also exhibit favorable responses, particularly in response to the ES3 electrical stimulus, with GelMA showing slightly higher neurite expression percentages. In contrast, alginate consistently demonstrates a low ability to promote neurite expression, regardless of the electrical stimulus. The interaction between the hydrogel and electrical stimulus reveals interesting patterns when considering neurite length as the dependent variable. Collagen shows significant fluctuations in neurite length across electrical stimuli. Alginate maintains a lower neurite length compared to other hydrogels. PEGDA exhibits a notable peak at ES2 and a valley at ES3, while GelMA demonstrates an increase in neurite length with ES2 and ES3, with ES3 yielding the highest peak among all hydrogels. This inconsistent behavior could have its origin in surface or chemical phenomena, which were not considered in the present study, revealing the need to investigate these phenomena further to understand the nature of these events. However, this research reveals significant, unknown results that help understand and direct future research.

## Figures and Tables

**Table 1:** AFM measurements of topographical parameters.

	Collagen	GelMA	PEGDA	Alginate
R_a_ (nm)	11.4	1.3	1.2	1.6
RMS (nm)	14.61	1.6	1.6	2.0
Young’s modulus (MPa)	0.96 ± 0.11	0.59 ± 0.21	4.10 ± 0.67	0.53 ± 0.03

## Data Availability

Data are contained within the article.
